# Nozzle tip damage in three generations of intraocular lens injector models: an experimental laboratory study

**DOI:** 10.1186/s12886-022-02726-y

**Published:** 2023-01-04

**Authors:** Lu Zhang, Sonja Schickhardt, Patrick Merz, Gerd Auffarth

**Affiliations:** Department of Ophthalmology, David J Apple Center for Vision Research, University Hospital Heidelberg, Im Neuenheimer Feld 400, Heidelberg, 69120 Germany

**Keywords:** Damage of nozzle tip, Intraocualr lens (IOL) injector, Johnson &Johnson

## Abstract

**Purpose:**

To assess the nozzle tip damage of IOL injectors in three generations from the same manufacturer using the self-developed system—the Heidelberg Score for IOL Injector Damage.

**Setting:**

David J Apple Center for Vision Research, Department of Ophthalmology, University Hospital Heidelberg, Heidelberg, Germany

**Design:**

Experimental laboratory study

**Methods:**

The nozzle tip damage of three injector models (Emerald, iTec, and Simplicity) was determined using the Heidelberg score for IOL injector damage. Damage to the nozzle tip was examined under a microscope and graded as follows: no damage (score 0), slight scratches (1), deep scratches (2), extensions (3), cracks (4) and bursts (5). The total scores for each injector system were the sum of scores for all injectors in this model. Total scores of the three injector systems were evaluated and compared. The nozzle tip parameters (diameters, tip angles) were also measured in each group.

**Results:**

The Emerald system achieved the highest total scores, while the other two systems achieved similar total scores. There was no statistically significant difference in the total scores between the study groups (*P* > 0.05). The outer cross-sectional diameters were 2.10 and 2.10 mm for Emerald, 1.80 and 1.78 mm for iTec, and 1.78 and 1.80 mm for Simplicity. The thickness of the nozzle tips was 0.13 mm (Emerald), 0.17 mm (iTec) and 0.17 mm (Simplicity). The tip angle for three injector models was 35° (Emerald), 45° (iTec), and 45° (Simplicity).

**Conclusions:**

Although different injector models exhibited varying degrees of damage to the nozzle tip, all injector models generally showed relatively good results. Newer generations of IOL injector models tend to perform better in terms of nozzle tip damage after IOL implantation.

**Supplementary Information:**

The online version contains supplementary material available at 10.1186/s12886-022-02726-y.

## Key summary points

### Why carry out this study?

- ❿ The nozzle tip damage of IOL injectors have been documented and such damage might be associated with several complications during IOL implantations.
- ❿ Evaluation of nozzle tip damage in different generations of IOL injectors could provide insight into optimization in the nozzle tip design and smoother IOL implantations.

### What was learned from the study?

- ❿ Newer generations of IOL injector models tend to perform better in terms of nozzle tip damage after IOL implantations.
- ❿ The extent of nozzle tip damage may be influenced by the parameters and configurations of the nozzle tips, the injection method (preloaded or manually loaded), and the materials of the injector rods.

## Introduction

As modern cataract surgery has evolved, so has the intraocular lens (IOL) injector systems. In the very beginning, surgeons or theater staff had to manually load the IOL into the cartridge and assemble a cartridge onto a handpiece. The injection method was to screw the plunger forward to implant the IOL. Later, preloaded IOL injectors were available commercially. The injection method now could be screw or push. The advantages of preloaded IOL injectors include elimination of manual setting variability, avoidance of potential IOL loading errors and damage, reduced surgical time duration, cost and complexity, as well as reduced risk of contamination of instruments with microorganisms or other foreign bodies [1–4]. Manufacturers have undoubtedly played an important role in the development of IOL injectors and have continued to introduce new generations of IOL injectors to the market.

The nozzle tip is the end part of the IOL cartridge and where the IOL exits the cartridge. During an IOL implantation, the IOL enters the eye through the nozzle tip. The forces between the IOL surface and the nozzle tip, and between the plunger and the nozzle tip could cause damage to the nozzle tips. The diameters of the inner cross-sectional surface of each nozzle tip determine the space through which the IOL passes. If this space is too small, greater friction between the IOL and the inner walls of the injector nozzle can be expected, resulting in greater damage to the nozzle tip [5]. The thickness of the nozzle tip is determined by the difference between the inner cross-sectional diameter and the outer cross-sectional diameter. If the thickness is too small, the probability of a crack in the tube may increase. If the thickness is too large, the overall diameter of the nozzle tip would also increase, resulting in a larger corneal incision size. Therefore, it is important to understand the correlations between the damage of the nozzle tip and the diameters of the nozzle tip.

Quite a few studies have associated the IOL surface abnormalities after IOL implantation with cartridge damage. A previous study by Kleimann et al. [6] found that deposits on the IOL surface were seen only in IOLs with cracks on the cartridges, whereas no deposits were observed on the IOL surface in IOLs with undamaged cartridges. Similarly, in a study by Faschinger [7], the author found IOL surface abnormalities corresponded to the defects on the inner walls of the injector cartridge. Marcovich et al. [8] also considered the deposits on the IOL surface was probably the inner part of the cartridge and some of these surface abnormalities could persist for over a year and may cause subsequent complications in the eye. Thus, it is important to further investigate the damage to the injector cartridges, which might shed light on how to reduce IOL abnormalities.

Damage to the nozzle tips after IOL implantations may also indicate over-riding of the plunger. Singh *et al*. noted in their study [9], in each case of a cracked cartridge, there was evidence of plunger overriding the optic edge. Investigation of post-implantation injector damage could optimize the positioning of the IOL in the injector during the preloading process to ensure safer IOL implantation by manufacturers.

To assess whether the newer generation of IOL injectors has improved in terms of nozzle tip damage, it would be beneficial to evaluate IOL injectors of different generations from the same manufacturer. To our knowledge, no study has been performed to assess nozzle tip damage in IOL injectors of different generations from Johnson & Johnson. In our previous study, we presented our self-developed damage scale, the Heidelberg Score for IOL Injector Damage ("HeiScore"), which systematically assessed and compared four generations of IOL injectors [5]. Therefore, in this study, nozzle tip damage of three generations of Johnson & Johnson IOL injectors was assessed using the Heidelberg Score for IOL Injector Damage ("HeiScore").

## Material and methods

### IOL injector models

The tested articles were summarized in Supplemental Table 1.

Emerald cartridge with Unfolder handpiece, a screw-style injector model, was introduced in 2011. The metal handpiece and the plastic cartridge need to be manually loaded by surgeons or theatre staffs before use.

iTec, a preloaded injector model, was introduced in 2015. It is a screw-style injector model.

Simplicity, a preloaded injector model, was introduced in 2019. It is a screw-style injector model.

### Collection of IOL injectors

**Fig. 1:**
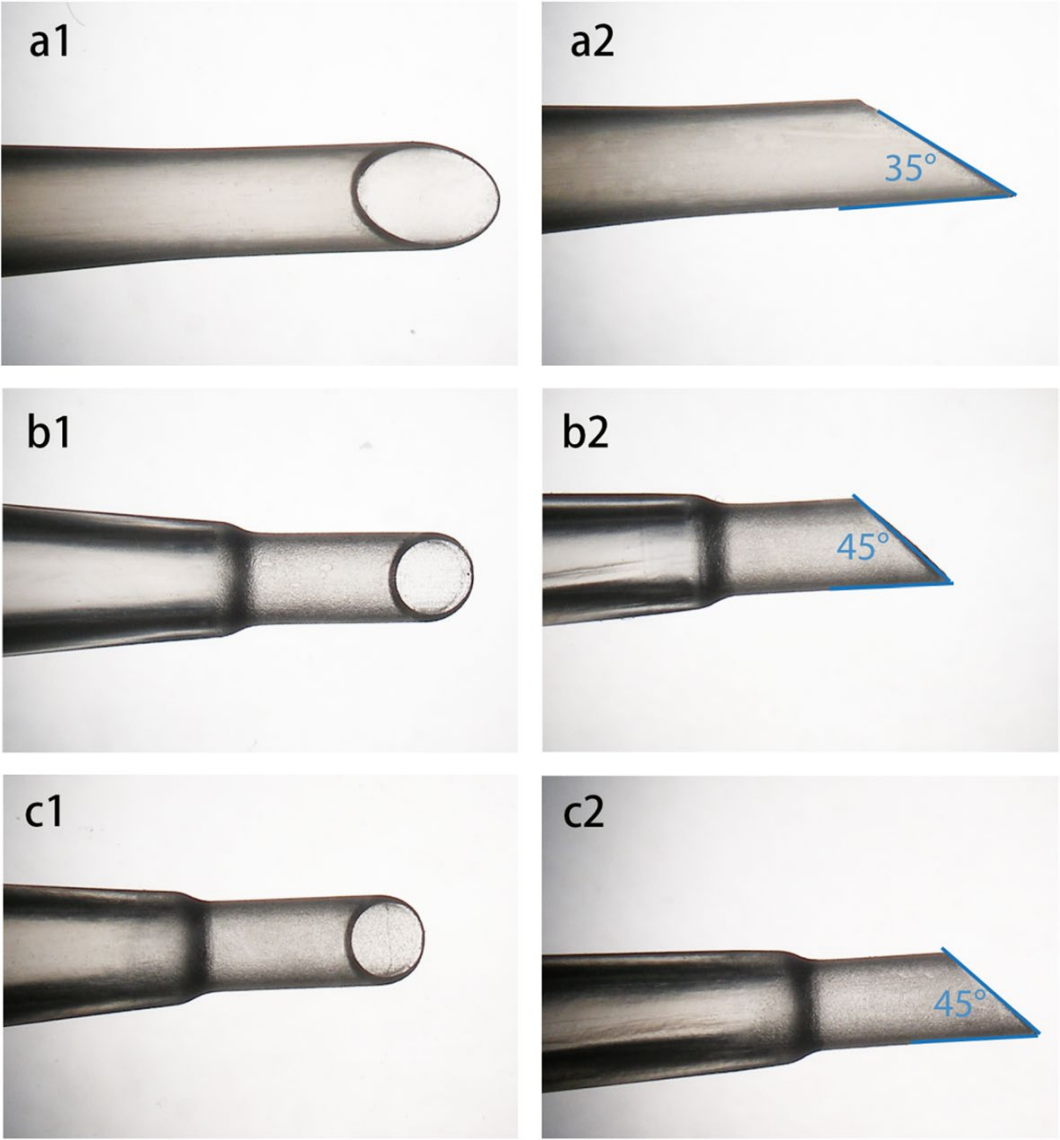
Representative microscopic images of three unused IOL injector models. a1&a2. Axial view and profile view of Emerald cartridge. b1&b2. Axial view and profile view of iTec. c1&c2. Axial view and profile view of Simplicity. Tip angels were marked in each injector model

Collection of the injectors was performed in the same manner as described in our earlier study [5]. Fifty-nine IOL injectors of three models were used for IOL implantation in a series of routine, uncomplicated cataract surgeries at the Heidelberg University Eye Hospital. One experienced surgeon (GUA) performed all the surgical operations. The microscopic image for one unused IOL injector from each model was shown in Fig. 1. Incisions were either 2.4 mm or 2.5 mm and were all clear corneal incisions. The range for IOL power was from + 15D to + 26D. In all cases, the injectors were first primed with 1% ophthalmic viscosurgical device (OVD). At the end of each implantation, a gross examination was performed under the microscope to determine whether the IOL had been damaged. After each surgery session, the used injectors were collected from the operation room and sent to our laboratory. Nozzles were immersed in the distilled water for ten minutes to remove the residual OVD and then dried by the air. Attention was paid not to damage the tips of the injector nozzles while handling.

### Damage evaluation of the nozzle tips

After air drying, the nozzles were examined using an optical microscope (Olympus BX50, Olympus K.K.). Each nozzle tip was first inspected in the "bevel down" and "bevel up" orientations, followed by examination of the two lateral orientations to identify any damage to the nozzle tip. Photographs of damage were then taken under microscope.

### Measurement of the nozzle tip parameters

**Fig. 2:**
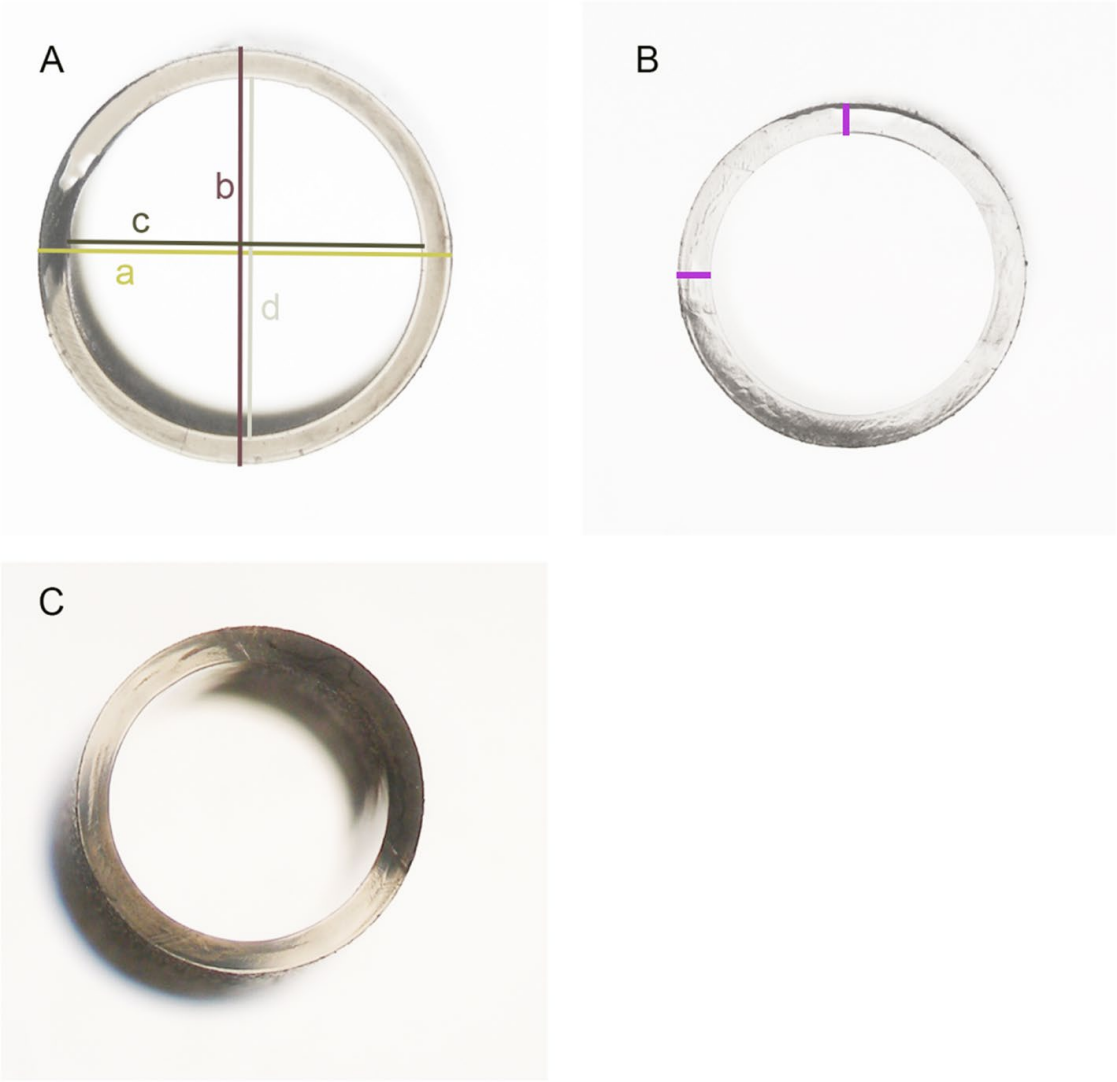
Representative microscopic images of cross-sectional surfaces for all injector models. **A** Emerald cartridge. **B** Simplicity. **C** iTec. a = outer cross-sectional horizontal diameter, b = outer cross-sectional vertical diameter, c = inner cross-sectional horizontal diameter, d = inner cross-sectional vertical diameter. Purple lines marked the thickness of the nozzle tip

To obtain a cross-sectional surface for each injector model, the nozzle tip of each unused injector model was cut with a razor blade at the point where the bevel angle began. Photographs of the cross-sectional surfaceswere taken under a microscope (Olympus BX50, Olympus K. K.). The parameters (i.e., diameters) of the cross-section surface were measured from an IOL photograph taken under the microscope at the same magnification as the cross-sectional surface, which served as a standard for calibrating the measurements. Image J software (version 1.52a, NIH, Bethesda, Maryland, USA) was used to measure the parameters of the cross-sectional surfaces of the nozzle tips. First, the known diameter of the IOL optics (6 mm) was taken as a reference, and the diameter on the image was measured in pixels. Second, the arithmetic ratio of pixels to millimeters was calculated. Third, the diameters of cross-sectional surfaces were measured on the image in pixels and then converted to millimeters using the arithmetic ratio. The inner and outer cross-sectional areas were calculated using the formula: A = πab (a = cross-sectional horizontal radius, b = cross-sectional vertical radius). The tip angles of three injector models were measured using Image J software (Fig. 1). The thickness of the nozzle tip was determined as the difference between the outer cross-sectional radius and the inner cross-sectional radius and calculated according to the formula: $\mathrm{Thickness}=\frac{\left(\mathrm{a}1-\mathrm{a}2\right)-(\mathrm{b}1-\mathrm{b}2)}{2}$ (a1 = outer cross-sectional horizontal radius, a2 = inner cross-sectional horizontal radius, b1 = outer cross-sectional vertical radius, b2 = inner cross-sectional vertical radius). (As marked by the purple lines in Fig. 2).

### Heidelberg Score for IOL Injector Damage (HeiScore)

According to our scoring system [5], the damage observed on the injectors was classified into the following six grades.

- **Grade 0**: There is no damage observed on the nozzle tips.
- **Grade 1**: There is slight scratch—fine stress lines on the inner tube or/and slight discontinuity at the nozzle tips.
- **Grade 2**: There is deep scratch—deep stress lines on the inner tube or/and obvious discontinuity at the nozzle tips.
- **Grade 3**: There is extension of “deep stress line”, but the deep stress line does not reach the level of full thickness tube crack.
- **Grade 4**: There is crack—full thickness crack of the injector tubes.
- **Grade 5**: There is burst of the injector tubes.

Each damage grade was assigned a score from 0 to 5 (i.e., Grade 0 was assigned to score 0, Grade 5 was assigned score 5, etc.), and the total damage scores for each injector system was the sum of scores for all injectors in that model. The total damage scores for each injector system were calculated and compared.

### Statistical analyses

To determine whether the damage scores and diopters of the IOLs were normally distributed in each IOL group, the Saphiro-Wilks test was used. To examine significant differences in the total scores between different injector models, the Kruskal–Wallis H test with Dunn’s adjustment was used for post hoc comparison. To investigate significant differences in diopters of the IOLs between groups, one-way analysis of variance (ANOVA) with Tukey adjustment was performed. All statistical analyses were performed with GraphPad Prism (version 9.0, GraphPad Software, SD, USA), and a *P* value of less than 0.05 was considered statistically significant.

## Results

### Parameters of nozzle tips

Nozzle tip parameters for all injector models are summarized in Table 1. All three injector groups had a round cross-sectional surface. In Emerald, both the outer and inner cross-sectional diameters were relatively larger, while iTec and Simplicity had almost identical cross-sectional diameters. Representative microscopic images of cross-sectional surfaces for all injector models are shown in Fig. 2. Emerald showed a smaller nozzle tip thickness (0.13 mm), whereas the other 2 groups had the same nozzle tip thickness (0.17 mm). The tip angle for three injector models was: 35° (Emerald), 45° (iTec), and 45° (Simplicity).

**Table 1:** Nozzle tip parameters of three injector systems

**Injector Model**	**Outer Cross-sectional Horizontal Diameter (mm)**	**Outer Cross-sectional Vertical Diameter (mm)**	**Outer Cross-sectional Area (mm**^**2**^**)**	**Inner Cross-sectional Horizontal Diameter (mm)**	**Inner Cross-sectional Vertical Diameter (mm)**	**Inner Cross-sectional Area (mm**^**2**^**)**	**Thickness of the nozzle tip (mm)**
Emerald	2.10	2.10	3.46	1.85	1.82	2.64	0.13
iTec	1.80	1.78	2.52	1.45	1.45	1.65	0.17
Simplicity	1.78	1.80	2.52	1.45	1.46	1.66	0.17

### Distribution of damage profiles

**Fig. 3:**
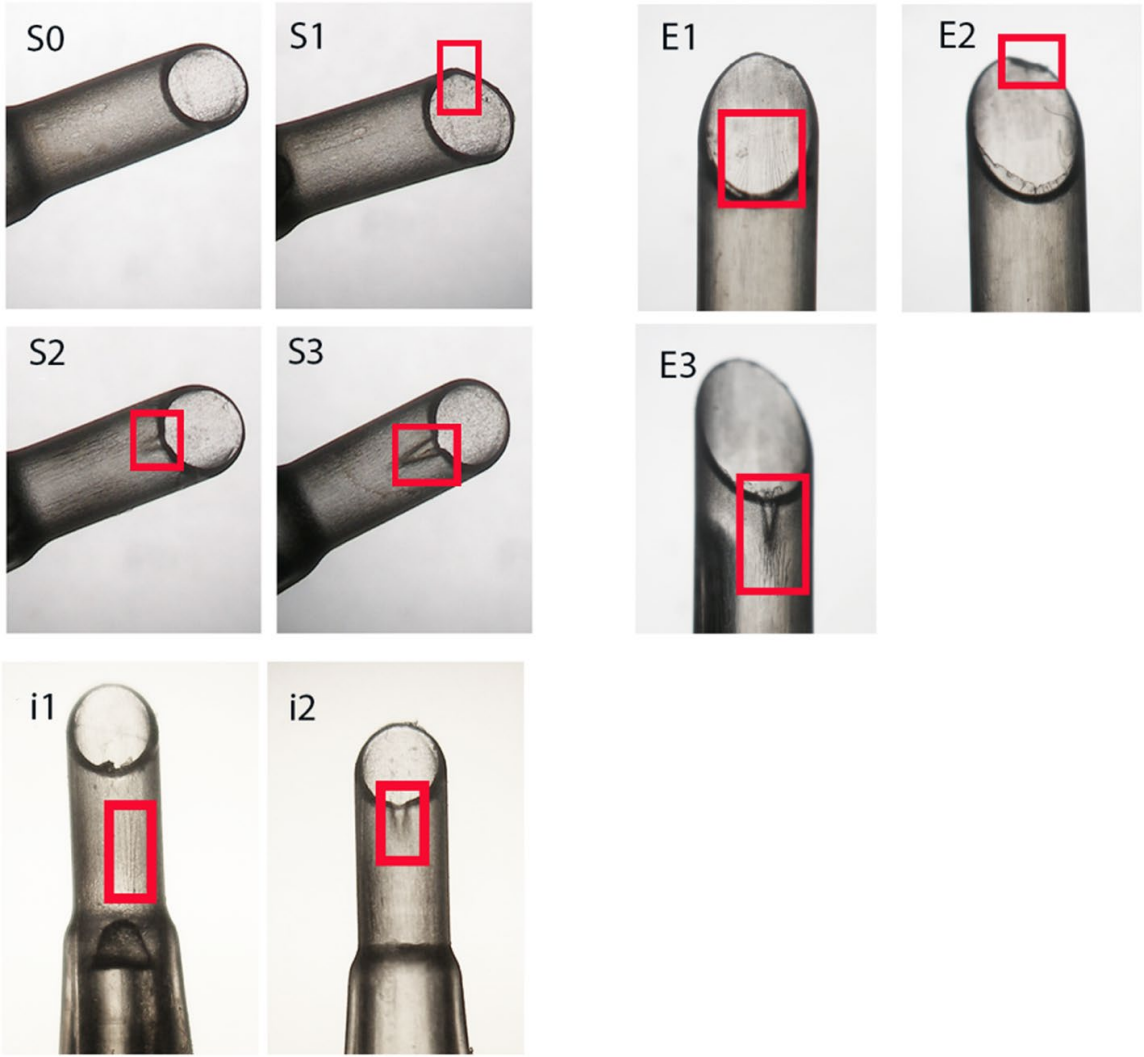
Representative microscopic images of each damage classification in each injector model. S = Simplicity, E = Emerald, i = iTec. S0. No damage. S1. Red square indicated slight discontinuity at the nozzle tip, graded as “slight scratches”. S2. Red square indicated obvious discontinuity at the nozzle tip, graded as “deep scratches”. S3. Red square indicates partial crack of tube, graded as “extension”. E1. Red square indicates fine stress lines on the inner tube, graded as “slight scratches”. E2. Red square indicated obvious discontinuity at the nozzle tip, graded as “deep scratches”. E3. Red square indicates partial crack of tube, graded as “extension”. i1. Red square indicated fine stress lines at the nozzle tip, graded as “slight scratches”. i2. Red square indicated obvious discontinuity at the nozzle tip, graded as “deep scratches”

**Fig. 4:**
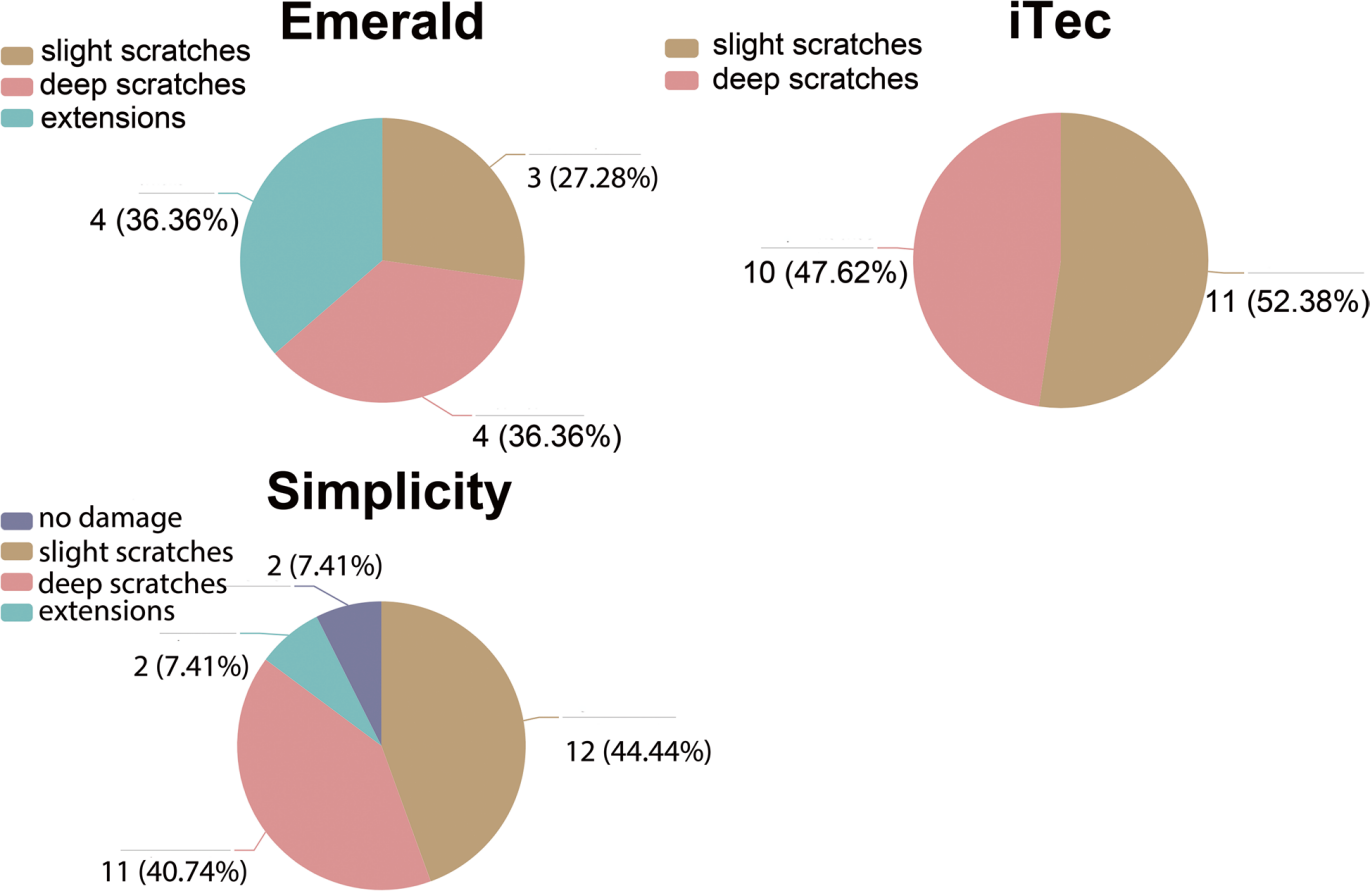
The distribution of damage profile in each injector model

Representative microscopic images of each damage classification in each injector group are shown in Fig. 3. The distribution of damage profiles of three injector models is shown in Fig. 4. iTec and Simplicity had a similar distribution of damage (mainly slight scratches and deep scratches). In Emerald, slight scratches (3, 27.28%), deep scratches (4, 36.36%), and extensions (4, 36.36%) were observed. For iTec, only slight scratches (11, 52.38%) and deep scratches (10, 47.62%) were observed. For Simplicity, no damage (2, 7.41%), slight scratches (12, 44.44%), deep scratches (11, 40.74%), and extension (2, 7.41%) were observed.

### Comparison between groups

**Fig. 5:**
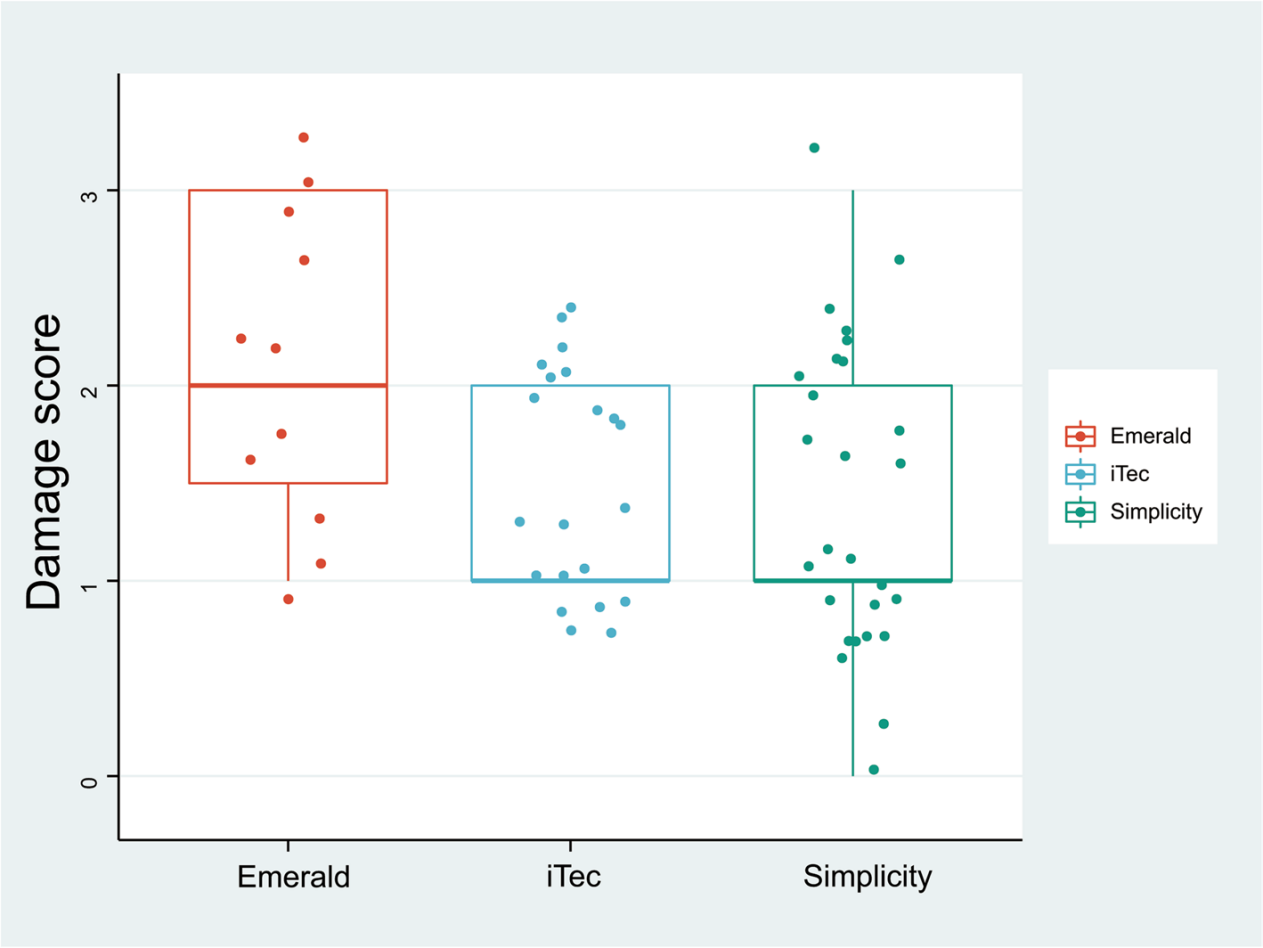
The damage assessment results for each IOL injector model

The damage assessment results for each IOL injector models are shown as boxplot diagrams (Fig. 5). The Emerald system yielded the highest damage score, while the other two injector systems presented similar damage scores. No statistically significant difference in the total score was found between the study groups (*P* > 0.05). Diopters of IOLs in each group were expressed as mean ± standard deviation (SD) (shown in Supplemental Fig. 1). No statistically significant difference was found between the study groups in terms of diopters of the IOLs (*P* > 0.05).

## Discussion

All IOLs were successfully implanted with the injectors in this study without damage to the capsular bag or other intraoperative complications. The IOLs were undamaged under gross microscopic inspection in the OR after each implantation. To our knowledge, this is the first study to systematically investigate the nozzle tip damage after IOL implantations in three generations of Johnson & Johnson IOL injector models.

In general, the nozzle tip damage after IOL implantation observed in our study was relatively mild and ranged from "no damage" to "extension." Severe damage like "crack" or "burst" was not found in any of the study groups. No statistically significant difference was found between the three groups in terms of damage scores. We suspect that since all three groups resulted in relatively low damage scores, far more samples would be required if there was a statistically significant difference to be detected. Although no statistically significant difference was found between the study groups in terms of IOL diopters of each IOL model, more standard deviations of diopters were found in the Simplicity group. This could be one reason why a more diverse distribution of damage category was observed in the Simplicity group. All three models were screw-style injector models. Compared with the push-style injector models, screw-style injection models require both hands to deliver the IOL into the eye. However, screw-style injection models may allow for more controlled and consistent IOL implantations, thereby avoiding complications such as "sudden IOL release." [10]. We speculate that this may be one reason why all three models in our study caused mild damage to the nozzle tips.

Compared to preloaded IOL injector models (iTec and Simplicity), manually loaded IOL injector model (Emerald) resulted in the highest damage score. This is consistent with one of our previous study [5] in which the manually loaded injector model generated a higher damage score than other preloaded injector models. We suggest that, on the one hand, preloaded injection models could provide more predictable injection and reduce complications during IOL implantations. On the other hand, the plunger of Emerald is metal, whereas the plungers of the other two injectors are plastic. The metal plunger could be a cause for the nozzle damage. Since stiffer than plastic, metal is more likely to scratch the inner walls than plastic plungers [5].

The diameters of the nozzle tip also play an important part in the extent of the nozzle tip damage. The thickness of the nozzle tip was quite similar for all injector models, suggesting that the thickness of the nozzle tip might not have an effect on the damage to the nozzle tip in this study. The inner cross-sectional area determines the space for an IOL to pass through during IOL insertion. If the space is too small, greater friction and higher range of damage to the injector nozzle tips could be anticipated [5]. Although the inner cross-sectional area of Emerald was the largest, the nozzle tip damage of Emerald was the greatest among the three injector models. This may be because, in addition to the diameters, the tip angle and the implantation method mentioned above (manually-loaded or preloaded), as well as the plunger material (metal or plastic), can also affect the extent of damage to the nozzle tips. Compared to iTec and Simplicity, Emerald had a more acute angle. Our result is consistent with a previous study by Nanavaty et al. [3] In Nanavaty's study, they concluded that the less acute the angle of the bevel tip, the less damage to the nozzle tip after IOL implantation. We are unable to confirm whether the quality of the nozzle tip materials have been improved from iTec to Simplicity. However, it appears that the nozzle tip configuration and parameters were almost identical for these two injector models. The fact that the damage scores and the distribution of damage categories were very similar for iTec and Simplicity can also be well explained by the identical configuration and parameters of these two injector models.

The limitation of this study is that we used IOLs with different diopters in each group. However, diopters of all the IOLs tested in our study ranged from + 15D to + 26D, which is the most common diopter range in clinical practice. In addition, no statistically significant difference was found between the three groups in terms of the dioptric power of the IOLs. Thus, when the injectors are manipulated according to the manufacturer's recommendations, the impact of the different diopters of the IOLs is negligible. Second, this study was simply a retrospective study, and the main purpose of the study was to show the different extent of damage to injectors of different generations and to compare the results. More studies are warranted to explore the correlation between the extent of injector damage and its clinical impact.

Although different injector models exhibited varying degrees of damage to the nozzle tip, all injector models generally showed favorable results. Newer generations of IOL injector models tend to perform better in terms of nozzle tip damage after IOL implantation. Further studies are needed to determine the relationship between the severity of injector damage and its clinical impact.

## Supplementary Information

**Additional file 1: Supplemental Table 1.** Test articles used in this study.

**Additional file 2: Supplemental Figure 1. **Diopters of IOLs in each group are expressed as mean ± standard deviation (SD).

## Data Availability

The data used to support the findings in this study are available from the corresponding author upon reasonable request.
