# Supplementary material for: Nozzle tip damage in three generations of intraocular lens injector models: an experimental laboratory study
Source: BMC Ophthalmol. 2023 Jan 4;23:7. doi: 10.1186/s12886-022-02726-y (PMC9811761; doi:10.1186/s12886-022-02726-y)
Supplement: Supplementary file 1 — Additional file 1: Supplemental Table 1. Test articles used in this study. [file 12886_2022_2726_MOESM1_ESM.docx]

Supplemental Table 1. Test articles used in this study

| **IOL model** | **IOL Injector model** |
| --- | --- |
| Sensar AR40e | UNFOLDER handpiece + Emerald cartridge |
| TECNIS PCB00 | iTec |
| TECNIS Eyhance DIB00 | Simplicity |
