# Supplementary figures and images for: Nozzle tip damage in three generations of intraocular lens injector models: an experimental laboratory study
Source: BMC Ophthalmol. 2023 Jan 4;23:7. doi: 10.1186/s12886-022-02726-y (PMC9811761; doi:10.1186/s12886-022-02726-y)

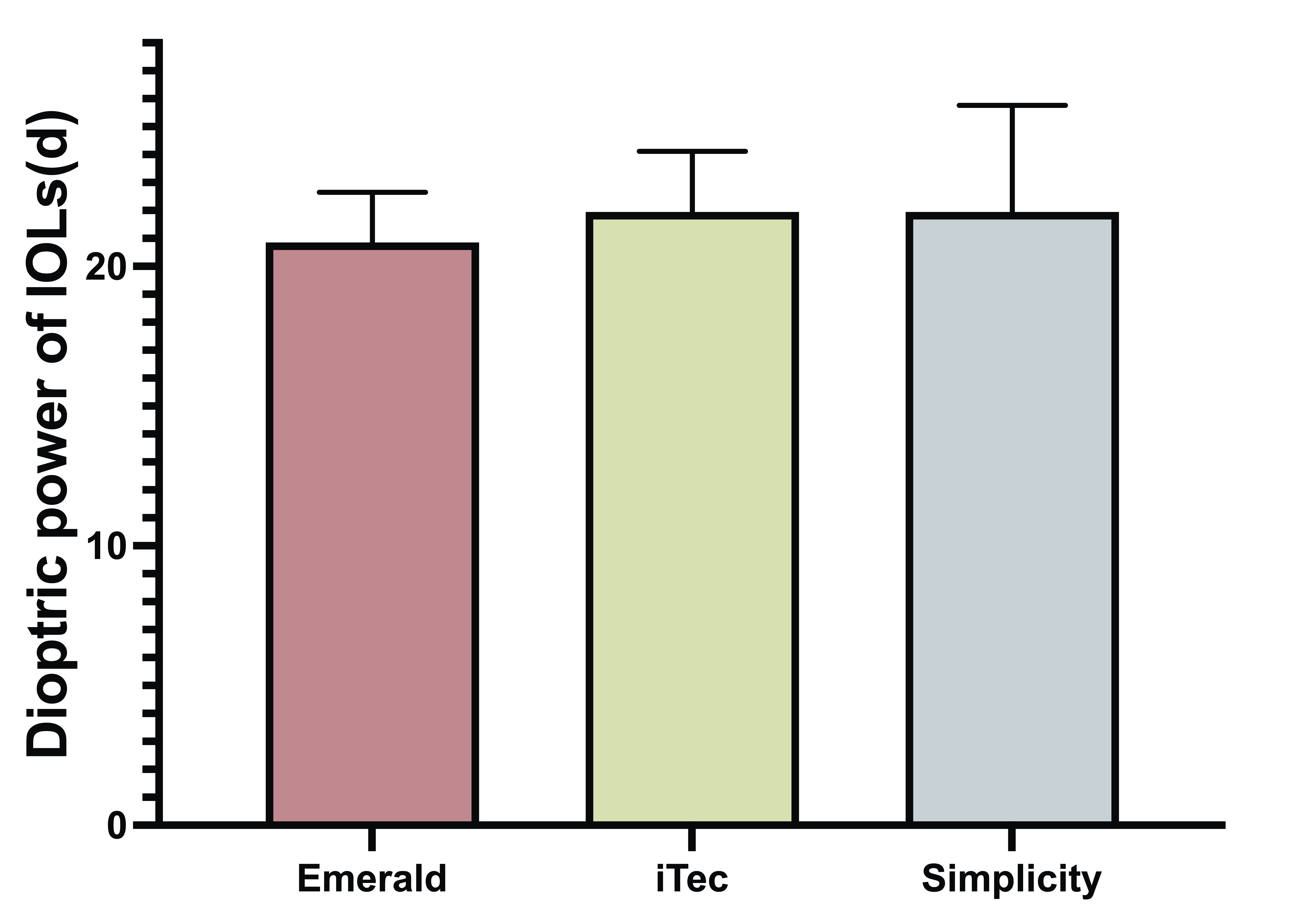

Supplement: Supplementary file 2 — Additional file 2: Supplemental Figure 1. Diopters of IOLs in each group are expressed as mean ± standard deviation (SD). [file 12886_2022_2726_MOESM2_ESM.tiff]
